# Supplementary material for: Associations of 24-hour movement guidelines adherence with fruit and vegetable intake in university students
Source: PeerJ. 2024 Aug 6;12:e17875. doi: 10.7717/peerj.17875 (PMC11313417; doi:10.7717/peerj.17875)
Supplement: Supplemental Information 3 [file peerj-12-17875-s003.pdf]

| Name | Label | Value | Labels |
|---|---|---|---|
| gender | sex | | |
| | | 1 | male |
| | | 2 | female |
| age | age | | |
| economic | | | |
| BMI | BMI | | |
| MVPA_guidelines | meeting the PA guidelines | | |
| | | 0 | not meeting |
| | | 1 | meeting |
| sleep_guidelines | meeting the sleep guidelines | | |
| | | 0 | not meeting |
| | | 1 | meeting |
| SB_guidelines | meeting the SB guidelines | | |
| | | 0 | not meeting |
| | | 1 | meeting |
| nutri2 | meeting the PA, SB, and sleep guidelines | | |
| | | 1 | never |
| | | 2 | sometimes |
| | | 3 | frequently |
| | | 4 | always |
| nutri3 | depression binary | | |
| | | 1 | never |
| | | 2 | sometimes |
| | | 3 | frequently |
| | | 4 | always |
